# Using fluorescence anisotropy to monitor chaperone dispersal of RNA-binding protein condensates

**DOI:** 10.1016/j.xpro.2022.101409

**Published:** 2022-05-18

**Authors:** Haneul Yoo, D. Allan Drummond

**Affiliations:** 1 Department of Biochemistry and Molecular Biology, The University of Chicago, Chicago, IL 60637, USA; 2 Department of Medicine, Section of Genetic Medicine, The University of Chicago, Chicago, IL 60637, USA

**Keywords:** Biophysics, Cell Biology, Molecular Biology, Protein Biochemistry

## Abstract

Heat stress triggers a specific set of proteins in budding yeast to form solid-like biomolecular condensates, which are dispersed by molecular chaperones. Here, we describe a protocol to study the kinetics of chaperone-facilitated condensate dispersal using biochemical reconstitution and fluorescence anisotropy. Although the current protocol is tailored to study heat-induced condensates of poly(A)-binding protein (Pab1), the protocol can be modified to study any protein which shows differential substrate binding activity upon condensation.

For complete details on the use and execution of this protocol, please refer to Yoo et al. (2022).

## Highlights

• Biochemical reconstitution of heat-induced Pab1 condensates
• Fluorescence anisotropy to monitor Pab1 condensate dispersal by molecular chaperones
• Data analysis to quantify the maximal rate of condensate dispersal

## Abstract (editor highlights)

Publisher’s note: Undertaking any experimental protocol requires adherence to local institutional guidelines for laboratory safety and ethics.

## Graphical abstract

**Figure:**
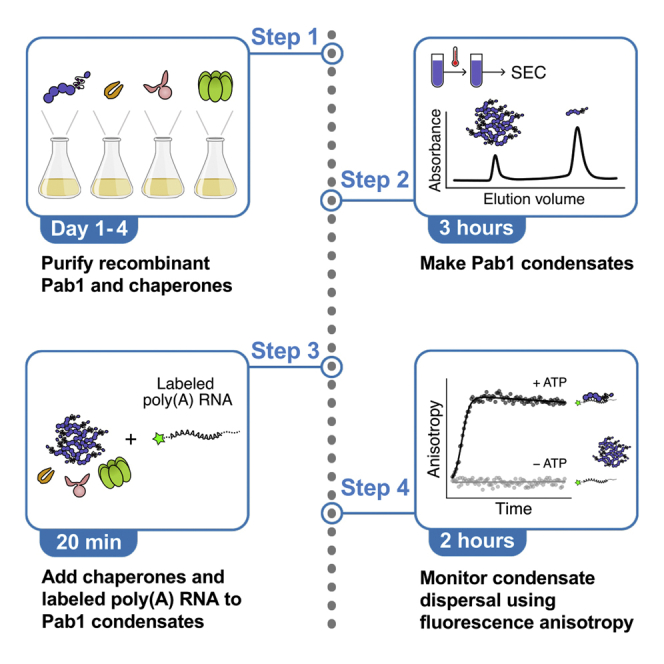

## Before you begin

This protocol describes the specific steps for using fluorescence anisotropy to monitor dispersal of heat-induced Poly(A)-binding protein (Pab1) condensates by the reconstituted yeast disaggregation system (Hsp104, Hsp70, Hsp40). Ssa2 and Sis1 are used as the sole Hsp70 and Hsp40, respectively. We have also used this protocol to test how the presence of additional co-chaperones and/or substrates affects the rate of Pab1 condensate dispersal (Yoo et al., 2022). To prepare the assay, we recombinantly express and purify *S. cerevisiae* Pab1, Hsp104, Ssa2, and Sis1 from *E. coli*. We use purified Pab1 monomers to make Pab1 condensates. Fluorescein-labeled 19-mer poly(A) RNA can be ordered from various custom oligonucleotide synthesis service providers including IDT and MilliporeSigma.

## Key resources table

**Table:** 

REAGENT or RESOURCE	SOURCE	IDENTIFIER
**Bacterial and virus strains**		

Subcloning Efficiency™ DH5α Competent Cells	Thermo Fisher Scientific	Cat#18265017
BL21(DE3) Competent Cells	MilliporeSigma	Cat#69450

**Chemicals, peptides, and recombinant proteins**		

SUPERase•In™ RNase Inhibitor	Thermo Fisher Scientific	Cat#AM2694
Creatine phosphokinase (CK)	MilliporeSigma	Cat#C3755
Creatine phosphate (CP)	GoldBio	Cat#C-323
Protease Inhibitor Cocktail Set IV	MilliporeSigma	Cat#539136
Pierce™ Protease Inhibitor Tablets, EDTA-free	Thermo Fisher Scientific	Cat#A32965
Adenosine-5′-Triphosphate (ATP)	GoldBio	Cat#A-081-1
Bovine serum albumin (BSA)	MilliporeSigma	Cat#12659

**Deposited data**		

Pab1 dispersal example data	This paper	Mendeley Data: https://doi.org/10.17632/4vzph2f83y.1

**Oligonucleotides**		

Fluorescein-A19 RNA: 5′ 6-FAM-AAAAAAAAAA AAAAAAAAA	IDT	N/A

**Recombinant DNA**		

Plasmid: 8×His-(TevC)-Pab1 in pET28a backbone	Riback et al. (2017)	pJAR006
Plasmid: 6×His-(SUMO)-Ssa2 in pET28a backbone	Yoo et al. (2022)	pHY024
Plasmid: 6×His-(SUMO)-Hsp104 in pET28a backbone	Yoo et al. (2022)	pEP055
Plasmid: 6×His-(SUMO)-Sis1 in pET28a backbone	Yoo et al. (2022)	pEP057

**Software and algorithms**		

RStudio ver. 1.2.1335	RStudio Team, 2018	http://www.rstudio.com/
SparkControl™ ver.	Tecan	N/A
Custom R (version 3.5.2) script for example data processing, analysis, and quantification	This paper	Mendeley Data: http://doi.org/10.17632/4vzph2f83y.1

**Other**		

HisTrap FF Column	Cytiva	Cat#17525501
HiTrap Heparin HP Column	Cytiva	Cat#17040701
HiTrap Q HP Column	Cytiva	Cat#17115401
Superose 6 10/300 GL Column	Cytiva	Cat#29091596
SnakeSkin Dialysis Tubing, 10K MWCO, 22 mm	Thermo Fisher Scientific	Cat#68100
NBS™ 384 well microplates (black; flat bottom) for anisotropy assays	Corning	Cat#CLS3575
Nunc™ Sealing Tapes for microplates	Thermo Fisher Scientific	Cat#235307

## Materials and equipment

### Plate reader setting

We used Spark microplate reader (Tecan) with monochromators. Excitation/emission wavelengths of 485/535 nm, each with a bandwidth of 20 nm, were used. G factor was calibrated with a solution of free 6-iodoacetamidofluorescein to produce a fluorescence polarization reading of 20 mP. Gain and Z-position were optimized with a 15 μL sample of 0.2 μM fluorescein-A19 RNA in 1× disaggregation buffer. We always set the internal temperature of the instrument to 30°C and wait until the temperature reaches 30°C before taking measurement. Fluorescence anisotropy was measured every 20 s for 1–2 h.

***Alternatives:*** We have been able to use A19 RNA labeled with ATTO550 using excitation/emission wavelengths of 550/585 nm, each with a bandwidth of 10 nm.

**Table:** 2× Disaggregation buffer

Reagent	Initial concentration	Final concentration	Amount
HEPES-KOH pH 7.3	0.5 M	40 mM	8 mL
KCl	2 M	300 mM	15 mL
MgCl_2_	0.5 M	5 mM	1 mL
Triton X-100	100%	0.02%	20 μL
ddH_2_O	n/a	n/a	56 mL
**Total**	**n/a**	**n/a**	**100 mL**

This reagent can be stored at 4°C or room temperature for up to one year. Filter buffer and avoid RNase contamination.

**Table:** 1× Disaggregation buffer

Reagent	Initial concentration	Final concentration	Amount
2× Disaggregation buffer	2×	1×	500 μL
BSA	10 mg/mL	0.5 mg/mL	50 μL
ddH_2_O	n/a	n/a	449 mL
DTT	1 M	1 mM	1 μL
**Total**	**n/a**	**n/a**	**1 mL**

Make this reagent fresh every time and do not store for longer than a day.

## Step-by-step method details

### Purification of recombinant Pab1, Hsp40, and Hsp104

**Timing: 4 days**

In this step, *S. cerevisiae* Poly(A)-binding protein (Pab1), Heat shock protein 104 (Hsp104), and type II Heat shock protein 40 (Hsp40/Sis1) are recombinantly expressed in *E. coli* and purified.

1. Express protein.
  a. Transform BL21(DE3) competent cells with a plasmid of interest.
    i. pJAR006, pEP055, and pEP057 plasmids have a kanamycin resistance cassette. Select the cells by plating them onto an agar plate with 1× kanamycin (50 μg/mL) and incubating the plate overnight for 12–18 h at 37°C.
  b. Prepare and autoclave 1 L Terrific Broth (TB) media for each protein of interest.
  c. Next day, pick a single colony and start a 5 mL Luria Broth (LB) overnight culture.
    i. Add 1× kanamycin (50 μg/mL) to each culture and grow the cells at 37°C.
  d. Next morning, use the overnight culture to inoculate a 1 L TB culture. Add 1× kanamycin (50 μg/mL) to the inoculated TB culture and grow the cells at 37°C with 250 rpm shaking.
  e. Move the culture to a 30°C incubator when absorbance at 600 nm reaches between 0.4 and 0.6. After 30 min, induce protein expression with 0.2 mM IPTG (Isopropyl β-D-1-thiogalactopyranoside).
  f. After 4–6 h of protein expression at 30°C, harvest cells by centrifuging the culture at 3,000 g for 15 min at 4°C.
  g. If proceeding immediately to protein purification, resuspend the cells in His binding buffer (20 mM HEPES pH 7.3, 150 mM KCl, 2.5 mM MgCl_2_, 20 mM imidazole, 10% glycerol, and 1 mM β-mercaptoethanol or BME) so that the total volume is 50 mL or less. Add a Pierce protease inhibitor tablet (1 tablet per 50 mL) listed in the key resources table. Otherwise, freeze the cell pellet in liquid nitrogen and store it at −80°C.
2. Protein purification day 1.
  a. Lyse cells by sonication in His binding buffer supplemented with a Pierce protease inhibitor table, as described in the previous step.
    i. An amplitude of 60% was used to sonicate the cells for 5 s followed by 5 s of pause, for the total pulse time of 20 min.
  b. Clear the lysate by centrifuging at 20,000 g for 15 min at 4°C.
  c. Take supernatant and load onto a 5 mL HisTrap column using an AKTA FPLC system and a 50 mL Superloop. Specific purification columns used in the original study are listed in the key resources table.
    ***Note:*** Other methods such as Ni resin and gravity columns would also work.
    i. Wash thoroughly with at least 4 column volumes (CV) of His binding buffer.
    ii. Elute with His elution buffer (His binding buffer with 400 mM imidazole).
  d. Pool elution fractions and dilute 2-fold using Q binding buffer (20 mM HEPES pH 7.3, 50 mM KCl, 2.5 mM MgCl_2_, 10% glycerol, and 1 mM Dithiothreitol or DTT) to reduce the salt concentration to around 100 mM.
    i. For Pab1, load the sample to a Heparin column to remove RNA. For Hsp104 and Hsp40, load the sample to a HiTrap Q anion exchange column.
    ii. Wash thoroughly with at least 4 CV of Q binding buffer.
    iii. Elute proteins from both Heparin and Q columns with a 20 mL gradient from 0 to 100% Q elution buffer (Q binding buffer with 1 M KCl).
  e. Pool elution fractions and add an aliquot of tobacco etch virus (TEV) protease (Tropea et al., 2009) to Pab1 or SUMO protease Ulp1 (Malakhov et al., 2004) to Hsp104/40 to remove the purification tag.
  f. Using a 10 kDa cutoff SnakeSkin dialysis tube, dialyze proteins with the appropriate protease overnight for 12–18 h at 4°C in 1 L of His binding buffer.
3. Protein purification day 2.
  a. To remove uncleaved protein, load the dialyzed sample through a HisTrap column and collect flow-through.
  b. Concentrate the sample using a spin concentrator and load the sample onto a size exclusion column equilibrated in storage buffer (20 mM HEPES pH 7.3, 150 mM KCl, 2.5 mM MgCl_2_, and 1 mM DTT).
  c. Pool fractions containing protein of interest, concentrate, and freeze aliquots in liquid nitrogen for storage at −80°C. Concentration was measured using Bradford assay with BSA standard.

### Purification of Hsp70

**Timing: 4 days**

In this step, *S. cerevisiae* Heat shock protein 70 (Hsp70) is recombinantly expressed in *E. coli* and purified.

***Note:****S. cerevisiae* has four cytosolic Hsp70 chaperones (Ssa1–4). Ssa1 and 2 are constitutively expressed while Ssa3 and 4 are induced upon stress. We specifically chose to work with Ssa2 because it is constitutively expressed and is the most abundant cytosolic Hsp70 in *S. cerevisiae* (Csárdi et al., 2015). However, we have also purified Ssa1 and Ssa4 using this protocol and used them to disperse Pab1 condensates in the original study (Yoo et al., 2022).

4. Express protein.
  a. Transform BL21(DE3) competent cells with a plasmid encoding 6×His-(SUMO)-Ssa2 (pHY024). This plasmid has a kanamycin resistance cassette. Select the cells by plating them onto an agar plate with 1× kanamycin (50 μg/mL) and incubating the plate overnight for 12–18 h at 37°C.
  b. Prepare and autoclave 2 L TB media.
  c. Next morning, pick multiple colonies and use them to start a 25 mL LB culture with 1× kanamycin. Grow the cells at 37°C with 250 rpm shaking until absorbance at 600 nm reaches between 0.4 and 0.6.
  d. Inoculate each of the 1 L TB media with 10 mL of the starter LB culture. Add 1× kanamycin to the TB cultures. Grow the cells at 37°C with 250 rpm shaking.
  e. Move the cultures to an 18°C incubator when absorbance at 600 nm reaches between 0.4 and 0.6. After 30 min, induce protein expression with 0.2 mM IPTG.
  f. After 14–18 h of protein expression at 18°C, harvest cells by centrifuging for 15 min at 3,000 g, at 4°C.

***Note:*** We recommend immediately lysing the cells and starting Hsp70 purification.

**Figure 1:**
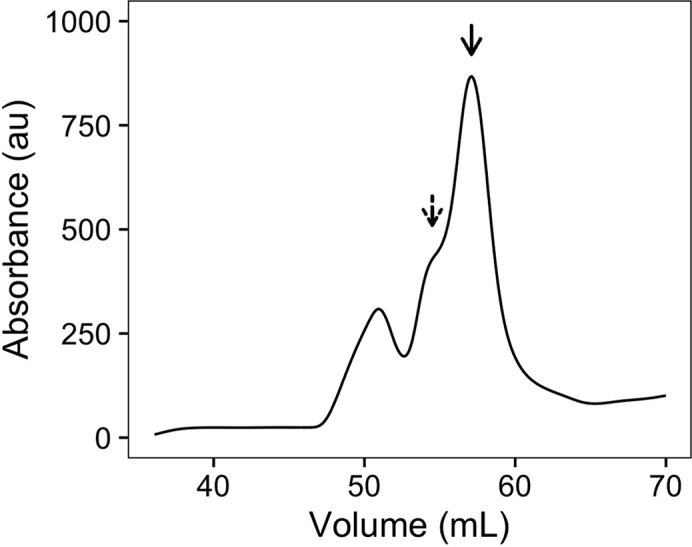
A representative elution profile of Hsp70 from the anion exchange column Hsp70 eluted in a peak around 25 mS/cm (solid arrow) with a left shoulder (dashed arrow). Only the elution fractions belonging to the main peak (solid arrow) were collected.
  d. Concentrate the protein if necessary and freeze aliquots in liquid nitrogen for storage at −80°C. Concentration was measured using Bradford assay with BSA standard.
    ***Note:*** Because the storage buffer contains 1 mM ATP, absorbance at 280 nm cannot be used to determine Hsp70 concentration.

5. Hsp70 purification day 1.
  a. Resuspend the cells in Hsp70 His binding buffer (50 mM HEPES pH 7.3, 750 mM KCl, 5 mM MgCl_2_, 20 mM imidazole, 10% glycerol, 1 mM BME, and 1 mM ATP) supplemented with a Pierce protease inhibitor tablet listed in the key resources table (1 tablet per 50 mL).
  b. Lyse cells by sonication.
    i. We used an amplitude of 60% for 5 s of sonication followed by 5 s of pause, for the total pulse time of 20 min.
  c. Clear the lysate by centrifuging at 20,000 g for 15 min at 4°C.
  d. Take supernatant and use a 5 mL HisTrap column to bind His-tagged Ssa2.
    i. Wash thoroughly with at least 5 CV of Hsp70 His binding buffer.
    ii. Equilibrate the column with Hsp70 His binding buffer supplemented with 20 mM ATP. Incubate the column and the bound protein in this high ATP buffer for at least 30 min at 4°C to saturate Hsp70 with ATP and promote substrate release from Hsp70.
    iii. Wash the column again with at least 4 CV of Hsp70 His binding buffer.
    iv. Elute Hsp70 with Hsp70 His elution buffer (Hsp70 His binding buffer with 400 mM imidazole).
  e. Pool elution fractions and use a 10 kDa cutoff SnakeSkin dialysis tube to dialyze the sample against 1 L Hsp70 binding buffer for at least 2 h at 4°C to reduce imidazole concentration. High imidazole concentration can inhibit protease activity.
  f. Add an aliquot of SUMO protease Ulp1 to the dialysis bag.
  g. Dialyze overnight for 12–18 h at 4°C in a 1 L Hsp70 His binding buffer.
6. Hsp70 purification day 2.
  a. Load the dialyzed sample through a HisTrap column and collect flow-through.
  b. Reduce salt concentration to below 100 mM by repeatedly concentrating and diluting the sample in Hsp70 Q binding buffer (20 mM HEPES pH 7.3, 50 mM KCl, 5 mM MgCl_2_, 0.5 mM EDTA, 2 mM DTT, and 1 mM ATP).
    i. Load the sample to a HiTrap Q anion exchange column.
    ii. Elute Hsp70 over a 50 mL gradient from 0 to 100% Hsp70 Q elution buffer (Hsp70 Q binding buffer with 1 M KCl). Troubleshooting 1.
      **CRITICAL:** Hsp70 often elutes in a broad single peak with a left shoulder or sometimes in two separate peaks around 25 mS/cm (Figure 1). Both peaks contain Hsp70, but only the later peak fractions exhibit activity in luciferase reactivation and Pab1 condensate dispersal assays. We recommend saving all elution fractions from this step and testing Hsp70 activity against Pab1 condensates and/or luciferase aggregates (Glover and Lindquist, 1998; Nillegoda et al., 2017) in each fraction before combining fractions. This step can be done once to optimize the protocol and skipped in subsequent preparations.
  c. Pool fractions containing active Hsp70 and buffer exchange into Hsp70 storage buffer (50 mM HEPES pH 7.3, 150 mM KCl, 5 mM MgCl_2_, 10% glycerol, 2 mM DTT, and 1 mM ATP).

### Preparation of Pab1 condensates

**Timing: 3 h**

In this step, we make small Pab1 condensates by exposing purified Pab1 monomers to the same heat shock condition that we use to stress yeast (Yoo et al., 2022). Upon exposure to heat, Pab1 undergoes autonomous demixing to form stable condensates (Riback et al., 2017). We isolate small Pab1 condensates from the remaining monomers using size exclusion chromatography.

7. Pab1 monomers were buffer exchanged into aggregation buffer (20 mM HEPES pH 6.8, 150 mM KCl, 2.5 mM MgCl_2_, and 1 mM DTT) and diluted to make a 500 μL sample of 25 μM Pab1.

***Note:*** This sample was sometimes supplemented with 0.25 μM firefly luciferase to increase yield (Yoo et al., 2022). About 30% of Pab1 elutes as condensates in the absence of luciferase and this increases up to 60% in the presence of luciferase.

8. Incubate the sample in a 42°C water bath for 20 min.
9. Centrifuge the sample for 3 min at 8,000 g at 4°C.

***Note:*** At pH 6.8, the sample remains clear after heat shock and no or minimal pelleting is observed.

**Figure 2:**
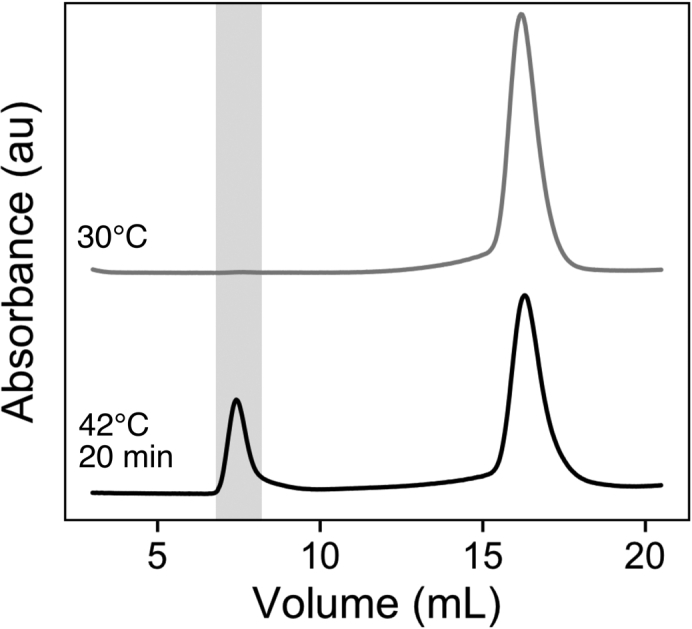
Representative size exclusion column traces for untreated (top) and heat shocked (bottom) Pab1 Pab1 condensates eluted in the void volume shaded in gray. The molecular weight cutoff for elution in the void volume is 5,000 kDa in Superose 6 10/300. The figure was adapted from Yoo et al. (2022) with permission from the authors.
12. Measure Pab1 condensate concentration in each fraction using Bradford assay with a BSA standard and/or SDS-PAGE with Pab1 monomer standards of known concentrations. Troubleshooting 2.

***Note:*** Typical Pab1 condensate concentration in a fraction ranges from 0.5 to 2 μM. Attempts to further concentrate condensates by dialysis or spin concentration led to sample loss.

10. Load the supernatant onto a Superose 6 10/300 size exclusion column equilibrated with storage buffer (20 mM HEPES pH 7.3, 150 mM KCl, 2.5 mM MgCl_2_, and 1 mM DTT).
11. Collect 0.25 mL fractions. Small Pab1 condensates elute in the void volume (Figure 2).

13. Freeze protein aliquots in liquid nitrogen and store at −80°C.

***Note:*** Although Pab1 condensates remain stable after thawing, we recommend making small aliquots and avoiding repeated freeze-thaw cycles.

### Preparation and execution of Pab1 condensate dispersal reactions

**Timing: 3 h**

**Figure 3:**
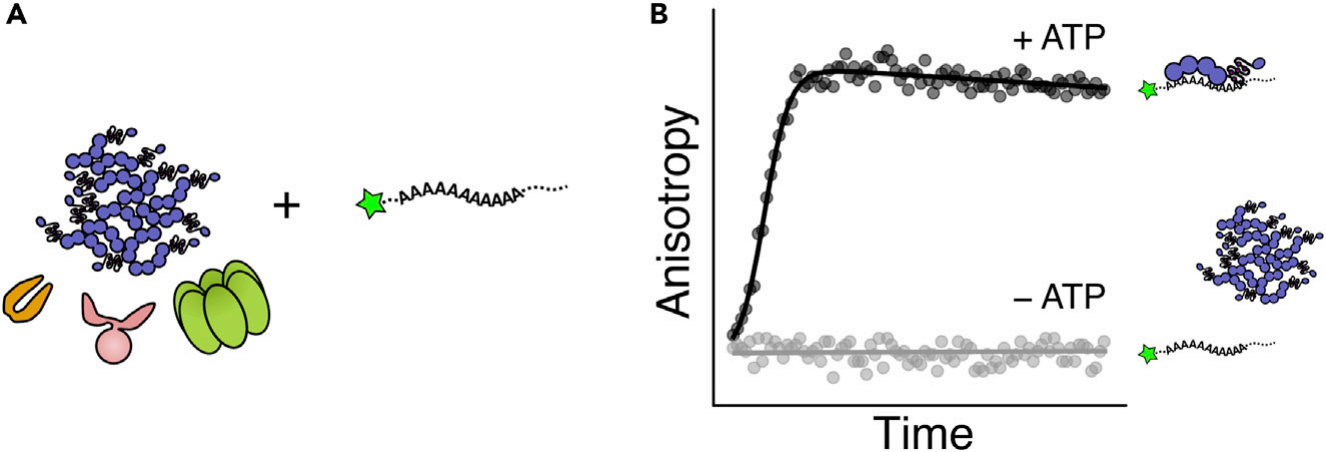
Overview of the dispersal assay (A) Pab1 condensates are mixed with molecular chaperones and fluorescein-labeled 19-mer poly(A) RNA. (B) Fluorescence anisotropy of the labeled RNA is measured as a function of time in the absence or presence of ATP. In the absence ATP, the labeled RNA remains unbound and this results in low fluorescence anisotropy. In the presence of ATP, chaperones disperse Pab1 condensates back to Pab1 monomers, which then bind the labeled RNA and cause an increase in fluorescence anisotropy.

In this step, we mix varying concentrations of Pab1 monomers with a fixed concentration of labeled poly(A) RNA to get a calibration curve. We then mix Pab1 condensates with molecular chaperones, ATP, and labeled 19-mer poly(A) RNA to start the dispersal reaction (Figure 3A). Because Pab1 condensates have a much reduced RNA binding activity compared to Pab1 monomers, condensate dispersal is monitored by the change in fluorescence anisotropy of the labeled RNA (Figure 3B).

***Note:*** Pab1 binds 12-mer poly(A) RNA with full affinity and has a footprint of roughly 25 nucleotides (Sachs et al., 1987). We used 19-mer poly(A) RNA to promote 1:1 binding of Pab1 monomer to RNA.

14. Set up calibration samples as shown in the table below.
  a. The working stocks of Pab1, ATP, and RNA were kept on ice. The 1× disaggregation buffer and the mixed calibration samples were kept at room temperature.
  b. Make a master mix to minimize error. Each sample is 15 μL.
  c. Samples can be mixed in an Eppendorf tube and transferred to a well in a 384-well plate, or mixed directly in the well. Troubleshooting 3 and 4.

**Table:** Pab1 monomer calibration samples

Reaction	Pab1 final concentration (μM)	Pab1 working concentration (μM)	Pab1 (μL)	0.1 M ATP (μL)	5 μM RNA (μL)	1× disaggregation buffer (μL)
1	0.8	4	3	0.75	0.6	10.65
2	0.4	2	3	0.75	0.6	10.65
3	0.2	1	3	0.75	0.6	10.65
4	0.1	0.5	3	0.75	0.6	10.65
5	0.05	0.25	3	0.75	0.6	10.65
6	0.025	0.125	3	0.75	0.6	10.65
7	0.0125	0.0625	3	0.75	0.6	10.65
8	0	0	3	0.75	0.6	10.65
**Final concentration**	**n/a**	**n/a**	**n/a**	**5 mM**	**0.2 μM**	**n/a**

15. Set up Pab1 condensate dispersal reaction samples as shown in the table below.
  a. The working stocks of all proteins, ATP, RNA, SUPERase In RNase Inhibitor, and the ATP regeneration system (creatine kinase and creatine phosphate, or CK and CP) were kept on ice. The 1× disaggregation buffer was kept at room temperature.

**Table:** Pab1 condensate dispersal reaction samples

Reaction	2 μM Pab1 condensate (μL)	0.1 M ATP (μL)	5 μM RNA (μL)	1× disaggregation buffer (μL)	SUPERase-in (μL)	CK/CP mix (μL)	Hsp104/Ssa2/Sis1 mix (μL)
1	3	0	0.6	7.1	0.3	1	3
2	3	0.75	0.6	6.35	0.3	1	3
**Final conc.**	**“0.4 μM”**	**5 mM**	**0.2 μM**	**n/a**	**2%**	**1 μM/8 mM**	**0.1/1/0.5 μM**

***Note:*** Add the key component(s) last to minimize downtime. In this example, ATP is added last.

***Note:*** Pyruvate kinase (PK) and phosphoenolpyruvate (PEP) cannot be used as an ATP regeneration system in this assay because PK binds RNA.

***Note:*** We have noticed and confirmed using western blot that we lose about 50% of Pab1 condensates during transfer, likely due to adsorption to plastic surfaces. To compensate for this loss, we set up each reaction with twice the target concentration of Pab1 condensates. For example, in the table above we aim the final concentration of Pab1 condensates to be around 0.2 μM by beginning with 0.4 μM Pab1 condensates. The actual amount of Pab1 in each well can be estimated during data analysis using the calibration curve.

***Note:*** Concentrations of Hsp104 and Sis1 are in terms of hexamers and dimers, respectively.

16. Add 15 μL of 1× disaggregation buffer to a well as a blank buffer control.
17. Seal the wells with a low autofluorescence microplate sealing tape listed in the key resources table to prevent sample evaporation.
18. Measure fluorescence anisotropy of all samples using the setting described in materials and equipment. Troubleshooting 3–5.
19. When the measurement is done, save the data and take the plate out of the plate reader.

## Expected outcomes

**Figure 4:**
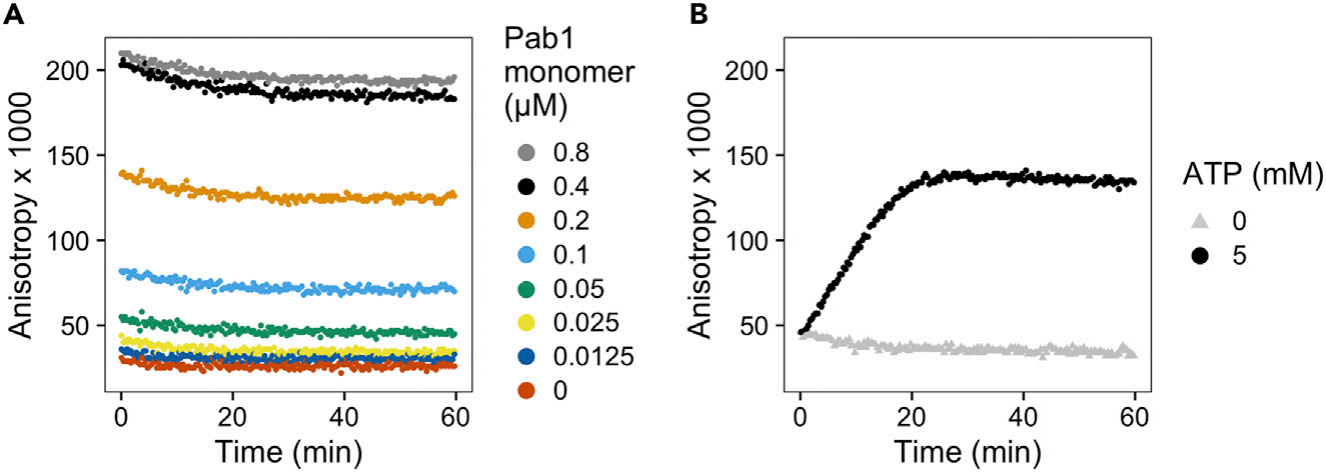
Expected unprocessed data (A) Calibration data collected over the duration of the experiment. (B) Pab1 condensate dispersal data. The slow decline in fluorescence anisotropy in the beginning is likely due to the increase in sample temperature to 30°C.

In Figure 4, the unprocessed calibration (Figure 4A) and kinetic data (Figure 4B) are plotted against time. See the next section for more detailed instructions on data processing and fitting.

## Quantification and statistical analysis

Here we further process the calibration data to extract fit parameters. These parameters are used to convert the y-axis of the kinetic data from fluorescence anisotropy to the concentration of RNA-bound Pab1 monomers. The kinetic traces are then fit with a logistic function to extract approximate rate of Pab1 condensate dispersal. Additionally, the y-axis of the raw kinetic trace can be converted to fraction Pab1 restored by background subtraction and normalization.

**Figure 5:**
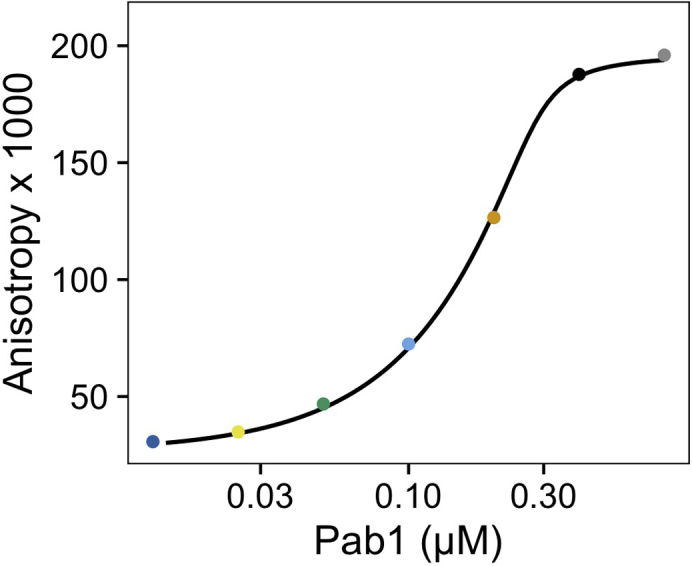
Calibration data plotted against Pab1 concentration Each data point is the mean of the corresponding trace from the whole time course in Figure 4A. The data are fitted with Equation 1.
  • The calibration data are fit with the following equation, where *min* and *max* refer to the minimum and maximum anisotropy values, *RNA* refers to the concentration of the labeled RNA, and *d* and *n* are the fit parameters.

1. Process the calibration data.
  • Compute the mean fluorescence anisotropy of each calibration sample and plot it against the concentration of Pab1 monomers (Figure 5).

(Equation 1) $$y=\min+\;\left(\max-\;\text{min}\right)\frac{\left(RNA+Pab1^{n}+d\right)-\sqrt{{\left(RNA+Pab1^{n}+d\right)}^{2}-4\left(RNA+Pab1^{n}\right)}\;}{2*RNA}\;$$

***Note:*** The concentration of the labeled RNA used in the assay is greater than the low nanomolar Kd of Pab1 to poly(A) RNA (Sachs et al., 1987). Quadratic binding equation did not give a good fit, suggesting we are operating under a titration regime (Jarmoskaite et al., 2020). Therefore, binding affinity cannot be determined from the calibration data. We use the calibration data and Equation 1, which is a modified quadratic binding equation, only to map a value of fluorescence anisotropy to a concentration of Pab1 monomer.

**Figure 6:**
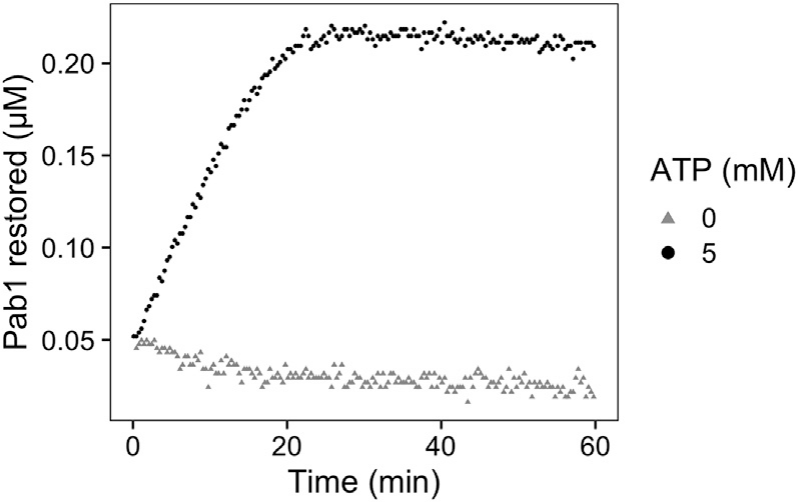
Pab1 condensate dispersal data after the conversion of the y-axis from fluorescence anisotropy to Pab1 monomer concentration Note that the reaction does not begin at zero due to the basal interaction between Pab1 condensates and RNA. Also note that the concentration of Pab1 at the plateau, i.e., after complete dispersal, is about half of the concentration of Pab1 we started with.
  • Fit the data with a logistic function (Equation 3) and compute the maximal rate of dispersal (Table 1) using the derivative of Equation 3 when *x* = *b* (Equation 4). The negative control was fitted with a linear model (Equation 5).
    (Equation 3) $$y=d+\frac{m}{1+e^{-a\left(x-b\right)}}-\;xc$$
    (Equation 4) $$rate_{max}=\frac{am}{4}-\;c$$
    (Equation 5) y=d−xc
    ***Optional:*** The raw kinetic trace can be normalized after background subtraction to convert the y-axis from fluorescence anisotropy to fraction Pab1 restored (Figure 7). This is assuming that all Pab1 is restored, which is confirmed using independent methods in the original study (Yoo et al., 2022). For the example data, we made two identical negative control samples and subtracted values from one of them from all samples. The same logistic and linear models (Equations 3 and 5) are used for fitting.

2. Extract the rate of Pab1 condensate dispersal.
  • Convert the fluorescence anisotropy values of the kinetic data to Pab1 concentrations (Figure 6) using the rearranged Equation 1 (Equation 2)*.*
    (Equation 2) $$Pab1=\sqrt[{n}]{\frac{{\left(y-\min\right)}^{2}\;\frac{RNA}{\left(\max-\;\min\right)}-\left(y-\min\right)\left(RNA+d\right)}{y-\max}}$$

**Table 1:** Fit parameters and the rate of dispersal extracted from the example data

ATP (mM)	d	a	b	C	m	Maximal rate (μM/min)
0	4.02e-2	0	0	3.19e-4	0	−3.19e-4
5	9.93e-4	1.793-e1	6.99	3.97e-4	2.31e-1	9.96e-3
    **Figure 7:**
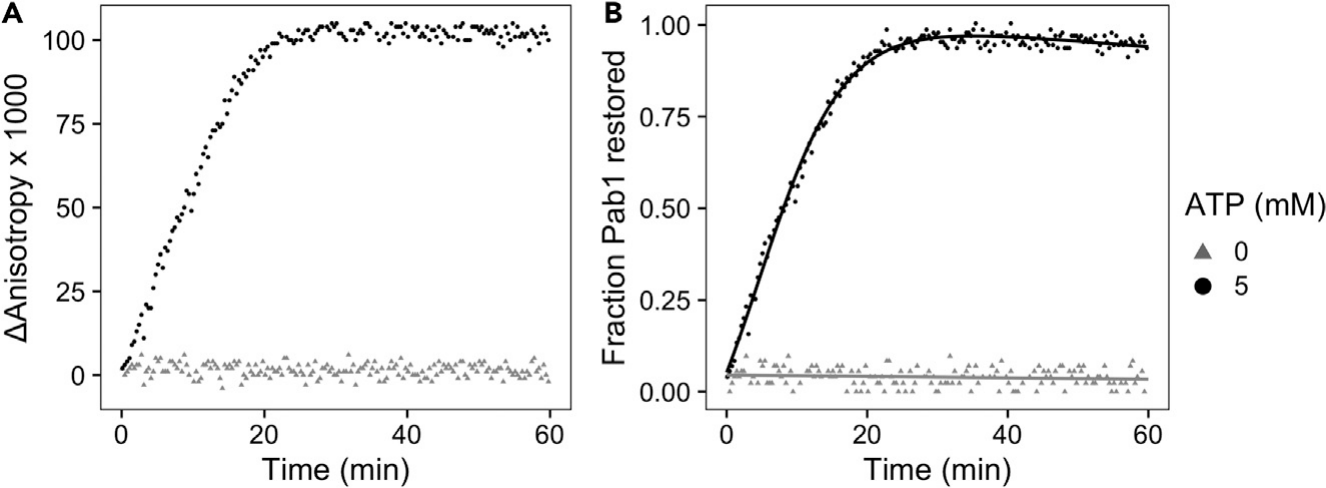
Pab1 condensate dispersal after background subtraction and normalization The y-axis of the dispersal data can be converted from fluorescence anisotropy to fraction Pab1 restored by performing (A) background subtraction and (B) normalization. The solid lines in (B) are the logistic and linear fits to the data.

## Limitations

**Figure 8:**
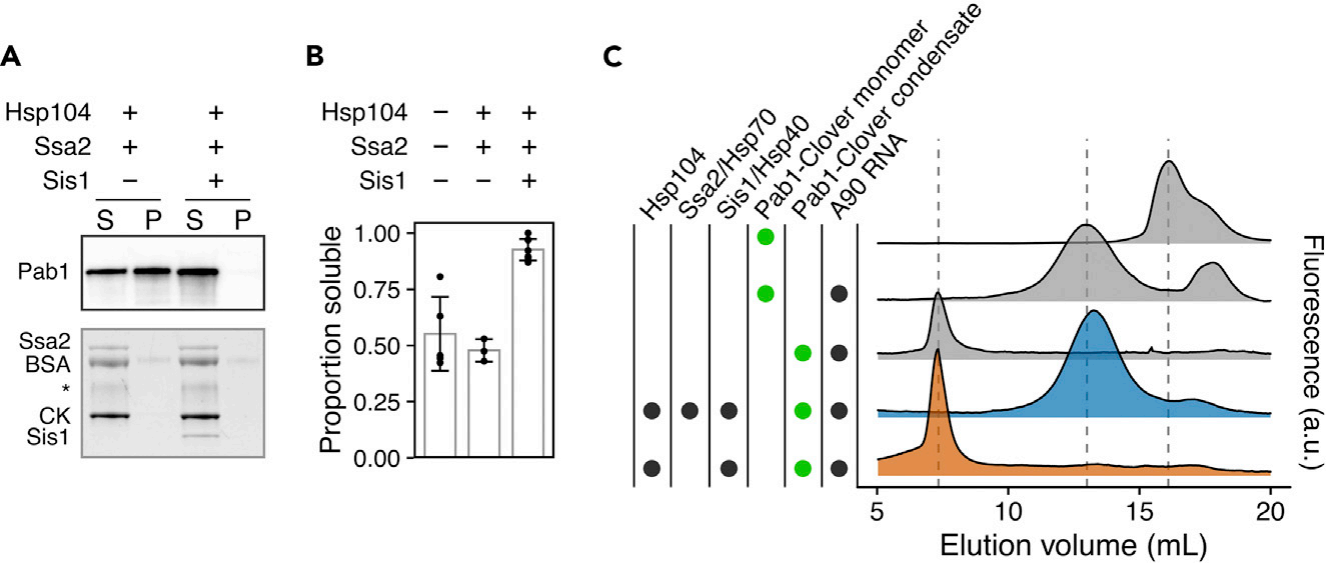
Example western blot and fluorescence-detection size exclusion chromatography (FSEC) data. (A) Western blot of Pab1 after sedimentation. Some small condensates remain soluble after centrifugation, but the pelletable Pab1 condensates disappear upon incubation with the same concentrations of chaperones and reagents used for the fluorescence anisotropy assay. Hsp104 is present at a low level and does not show up in the total protein gel shown at the bottom. Asterisk indicates unknown contaminant. (B) Quantification of Pab1 sedimentation results. Mean and standard deviation were computed from at least three experiments. (C) FSEC profiles of Pab1-Clover. The dashed lines, from left to right, mark the peaks corresponding to condensed, RNA-bound monomeric, and free monomeric Pab1-Clover. The data in this figure are adapted from Yoo et al. (2022) with permission from the authors. For preparation of A90 RNA, see Methods in Yoo et al. (2022).

This protocol relies on the fact that Pab1 condensates exhibit significantly reduced RNA binding activity compared to Pab1 monomers. Therefore, this protocol cannot be used to study proteins which show no or little difference in RNA binding activity upon condensation. Because Pab1 condensates exhibit substantially reduced but non-zero binding to RNA, the maximal rate of dispersal quantified from the fluorescence anisotropy data may be systematically underestimating the true rate of dispersal. To confirm that the end of the increase in anisotropy marks the end of condensate dispersal, we recommend analyzing the reaction samples by western blot or fluorescence-detection size exclusion chromatography (Figure 8), as done in the original study (Yoo et al., 2022).

## Troubleshooting

### Problem 1

Hsp70 protein purification yield is low (protocol step 6).

### Potential solution

Increase starting material to more than 2 L of TB. Make sure to use active Ulp1 protease during overnight dialysis. Sometimes too much protein induction can lead to protein aggregation and/or degradation. Try expressing protein for less time and/or using less IPTG.

### Problem 2

Condensate concentration is too low (protocol step 12).

### Potential solution

During condensate preparation, one can increase the input amount by heat shocking higher concentration of Pab1. Alternatively, one can heat shock Pab1 in the presence of a 100-fold less amount of thermolabile protein such as firefly luciferase to increase condensation yield (Yoo et al., 2022). Pab1 condensates seem to adsorb on to the plastic surfaces, leading to sample loss during transfer and pipetting. We recommend minimizing sample contact with plastic by removing unnecessary transfer steps. Avoid vortexing when mixing condensates with other components; gently pipet up and down or flicker the tube a few times and spin down the sample using a small tabletop centrifuge.

### Problem 3

Fluorescence anisotropy decreases in the calibration and/or in the reaction samples (protocol step 18).

### Potential solution

This is mostly likely due to RNase contamination in one of the reagents. Addition of commercial RNase inhibitors did not completely prevent the decay in signal. Identify, if possible, which reagent is contaminated and replace it with a fresh reagent. In our experience, recombinant Hsp70 is often the source of problem. Avoid taking Q column fractions contaminated with RNA-degrading factor(s). One can also lower the concentration of Hsp70 in the reaction to alleviate the problem.

### Problem 4

Fluorescence anisotropy signal is very noisy and unreliable (protocol step 18).

### Potential solution

Use a black 384 well microplate with non-binding surface to minimize RNA adsorption to the sides of the well. Having a little bit of detergent (for example, 0.01% Triton X-100) in the buffer also helps.

### Problem 5

Fluorescence anisotropy remains steady and does not increase in positive reactions (protocol step 18).

### Potential solution

First, check if the fluorescence anisotropy of reaction samples is comparable to that of the calibration sample with the target concentration of Pab1 monomers.

If the two values are comparable, one possibility is that the reaction happened too fast and completed before the measurement. Check condensate dispersal using orthogonal methods such as sedimentation and western blot and, if indeed all condensates have been dispersed, lower the concentrations of chaperones to slow the kinetics and/or optimize the workflow to reduce the downtime before measurement.

If the anisotropy values of the reaction samples are much higher, it is likely that one of the common reagents present in the reaction samples but not in the calibration samples is binding to RNA and saturating the signal. Make sure that none of the reaction reagents has RNA-binding activity. For example, pyruvate kinase and phosphoenolpyruvate cannot be used as the ATP regeneration system in this assay because pyruvate kinase binds to RNA.

If the anisotropy values of the reaction samples are much lower, it is likely that chaperones are not dispersing Pab1 condensates. Check the activity of the disaggregation system by performing luciferase disaggregation (Glover and Lindquist, 1998; Nillegoda et al., 2017).

## Resource availability

### Lead contact

Further information and requests for resources and reagents should be directed to and will be fulfilled by the lead contact, D. Allan Drummond (dadrummond@uchicago.edu).

### Materials availability

All unique and stable reagents generated in this study are available upon request.

## Data Availability

• The example data have been deposited to Mendeley Data: http://doi.org/10.17632/4vzph2f83y.1. The data are publicly available as of the date of publication. • The original R script used to process, analyze, and quantify the example data has been deposited to Mendeley Data: http://doi.org/10.17632/4vzph2f83y.1. The data are publicly available as of the data of publication. • Any additional information required to reanalyze the data reported in this paper is available from the lead contact upon request.
